# Role of gut microbiota metabolism and biotransformation on dietary natural products to human health implications with special reference to biochemoinformatics approach

**DOI:** 10.1016/j.jtcme.2022.03.005

**Published:** 2022-04-14

**Authors:** Mohd Hafizur Rehman Ansari, Sadia Saher, Rabea Parveen, Washim Khan, Imran Ahmad Khan, Sayeed Ahmad

**Affiliations:** a Bioactive Natural Product Laboratory, Department of Pharmacognosy and Phytochemistry, School of Pharmaceutical Education and Research, Jamia Hamdard, New Delhi, 110062, India; b Viral Research and Diagnosis Laboratory, Department of Microbiology, J.N.M.C, A.M.U, Aligarh, 202002, India; c Human Genetics Laboratory, Department of Bioscience, Jamia Millia Islamia, New Delhi, 110025, India; d Department of Chemistry, School of Chemical and Life Sciences, Jamia Hamdard, New Delhi, 110062, India

**Keywords:** Gut microbiota, Dietary natural compounds, Gut-microbial diversity, Mutualism strategies, GMM-GMBTs reactions, Bio-chemoinformatics, Gut microbiota-interplay disorders

## Abstract

Gut microbiota contributes to diverse mammalian processes including the metabolic functions of drugs. It is a potential new territory for drug targeting, especially for dietary natural compounds such as tannins, flavonoids, steroidal glycosides, anthocyanins, lignans, alkaloids, and others. Because most herbal medicines are orally administered, the chemical profile and corresponding bioactivities of herbal medicines may be altered and implication to ailments by specific microbiota through gut microbiota metabolisms (GMMs) and gut microbiota biotransformations (GMBTs). In this review, briefly introducing the interactions between different categories of natural compounds and gut microbiota produced countless microbial degraded or fragmented metabolites with their biological significance in rodent-based models. From natural product chemistry division, thousands of molecules are produced, degraded, synthesized, and isolated from natural sources but exploited due to lack of biological significance. In this direction, we add a Bio-Chemoinformatics approach to get clues of biology through a specific microbial assault to (Natural products) NPs.

## Graphical abstract

**Figure:**
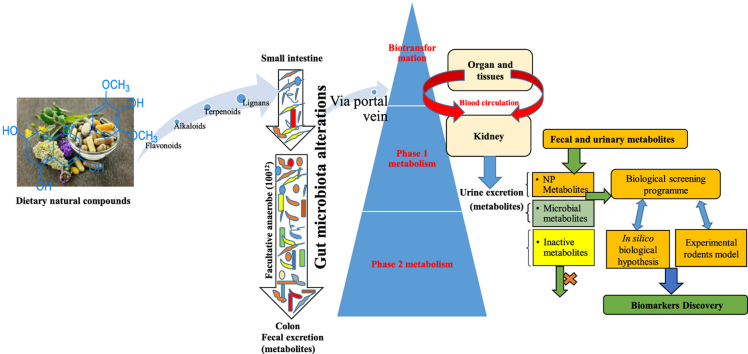

## 1 Introduction

**Fig. 1:**
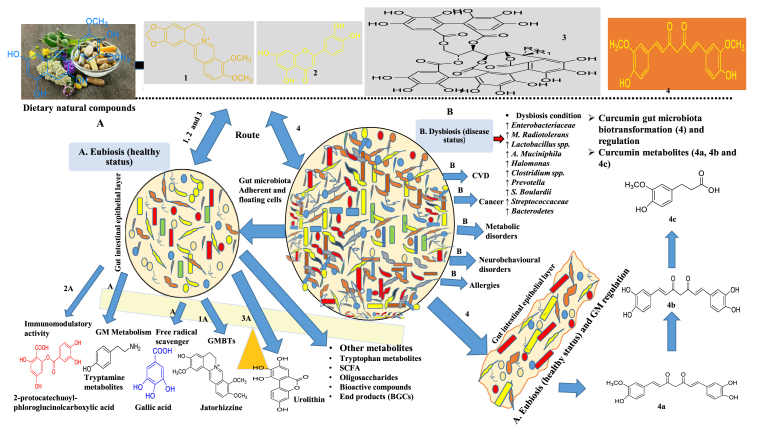
(A) Roles and modulation of gut microbiota. In addition transforming natural product metabolites i.e. biologically active regulates dysbiosis-eubiosis status. **(B)** Bidirectional interaction between curcumin and gut microbiota. Curcumin biotransformed by gut microbiota to more bioactive metabolites by several reactions than parent curcumin. After administration of curcumin, it regulate the gut microbiota by transformation of metabolites that gives alteration of microbial abundance, diversity and composition, which may exert health benefits via indirect mechanism.

The human gut is colonized by an extremely dense population of bacteria, collectively termed as microbiota or gut “flora” and is a site where they exert strong influences on human biology as well as drug fate.^1^^,^^2^ The entire system of human gut microbiota can be pictured as a microbial organ, which is closely associated with diverse processes including the metabolic function of drugs.^3^ Gut microbiota might be a potential new territory for drug targeting, especially for dietary (Natural products) NPs.^4^ A better understanding of the mechanisms of these interactions has led to greater interest in the corresponding secondary metabolites.^5^ Gut microbiota produces NPs in two distinct ways. Firstly, the metabolites are produced by gut microbiota from dietary components, such as tryptophan metabolites,^6^ short-chain fatty acids, oligosaccharides and, others. Secondly, the production of uncharacterized end products from the unique biosynthetic gene clusters (BGCs) of the gut microbiota. The past three decades have witnessed of great progress in BGC-derived natural product discovery from the mammalian gut microbiota. These NPs have potential for novel bioactive functions,^7^ such as the antibiotic microcin M/H47,^8^ the genotoxin colibactin,^9^ the cytotoxic compound tilivalline,^10^ and the protease inhibitors dipeptide aldehydes.^11^ These distinct secondary metabolites are played vital roles in understanding gut microbiota and in developing the pharmaceutical, agricultural, and food industries. As the concern of NPs, it shows a promising interplay host-microbiota functional interaction to the regulation of gut microbiota associated with several disorders like CVD, cancer, allergies, asthma, Crohn's disorder, and metabolic disorders mainly influenced under dysbiosis status. Pure compound curcumin gives eubiosis status and regulates gut microbiota-associated dysbiosis status from biotransformation with demethylation, hydrogenation, hydroxylation reactions as shown in (Fig. 1A and B).

Trillions of microorganisms colonize the human body, maintaining homeostasis and significantly influence human health and disease. These organisms are collectively known as the gut microbiota. It comprises many different bacterial species that are in dynamic, localized, close association with each other and with the host and play a vital role in the progression of several diseases. These microbiota are responsible for the extensive breakdown of the original dietary natural compounds into a low molecular weight metabolites, being absorbable, may actually be responsible for the health effects, rather than the original compounds. In total, the intestinal microbiota consists of approximately 500–1,000 species that, interestingly, belong to only a few of the known bacterial phyla with relative (%) abundance as shown in (Supplementary materials, Table 1). Gut bacteria can have beneficial effects such as the digestion of complex carbohydrates, colonization resistance against invading pathogens, maturation of the adaptive mucosal immune system, and the production of secondary metabolites, including vitamins. The evidence for host benefits comes both from our understanding of the metabolic services that the gut microbiota provides and studies of germ-free animal models.^12^ The ecosystem of the gastrointestinal tract is characterized by a great number of microbial species living in balance (eubiosis/dysbiosis) by adopting mutualistic strategies. Dysbiosis and chronic, subclinical, low-grade inflammation in the bowel wall contribute to and may even initiate the development of CRC, IBD to other extra-intestinal disorders^13^ as shown in (Supplementary materials, Table 2).

Some orally administered NPs by their alteration of chemical structures by GMMs and GMBTs to get into >1 fragmented metabolites, which exert better bioavailability and therapeutic effects than their parent compounds against ailments. Despite these NPs, others remain without biological significance. The components of these NPs regarding special computational biological screening through Bio-Cheminformatic tools are fewer reported. Three bioflavonoids such as indigocarpan, mucronulatol, indigocarpan diacetate, and two diterpenes namely erythroxydiol X and Y were derived from *I. aspalathoides* as PDGFRβ and VEGFR2 inhibitors nicely reported.^14^ Similarly, natural flavonoids and synthetic indole-chalcones were tested for *in silico* pharmacokinetic properties for validated as drug-like nature.^15^ Moreover, the analysis of therapeutic target for SARS-CoV-2 and discovery of potential drugs reported by computational methods.^16^ In the presented report, efforts have been made to discover and highlight strategies for further exploring the biosynthetic capacity of the human microbiome with distinct NPs. Besides, also briefly discusses the potential biological roles of these metabolites. From, Bio-Chemoinformatics approach that relies on *in silico* biological hypothesis with well-defined ADME protocol, which recommends for all probable degraded NP metabolites biopredictory role by the action of gut microbiota. At last, we reported some biology of NPs with gut microbiota modulation with the special context of biological disorders like cancer, NAFLD, GIP, metabolic, and neurobehavioral disorders. So, this report harnessing of NP metabolites predicted with *in silico* biological hypothesis. This tool protects the exploitation and makes use of degraded metabolites for the Natural Product Chemistry division for a further drug-discovery innovation program.

## 2 Gut microbiota functional transformation of NPs from GMM and GMBTS

Gut bacteria can hydrolyze glycosides, glucuronides, amides, esters, sulfates, and lactones. They undergo reduction, de-carboxylation, de-methylation, ring cleavage, and dihydroxylation reactions.^17^ The report aimed to summarize the current information about the microbial degradation metabolites and their formation pathways obtained from the different groups of dietary NPs, identifying their differences and similarities. Recent progress in the identification of colonic microbial species responsible for NPs and novel tools used to identify them and the impact of NPs microbial metabolism and biotransformation on their bioavailability and bioactivity were also reviewed.

### 2.1 Gut microbiota metabolism

**Fig. 2:**
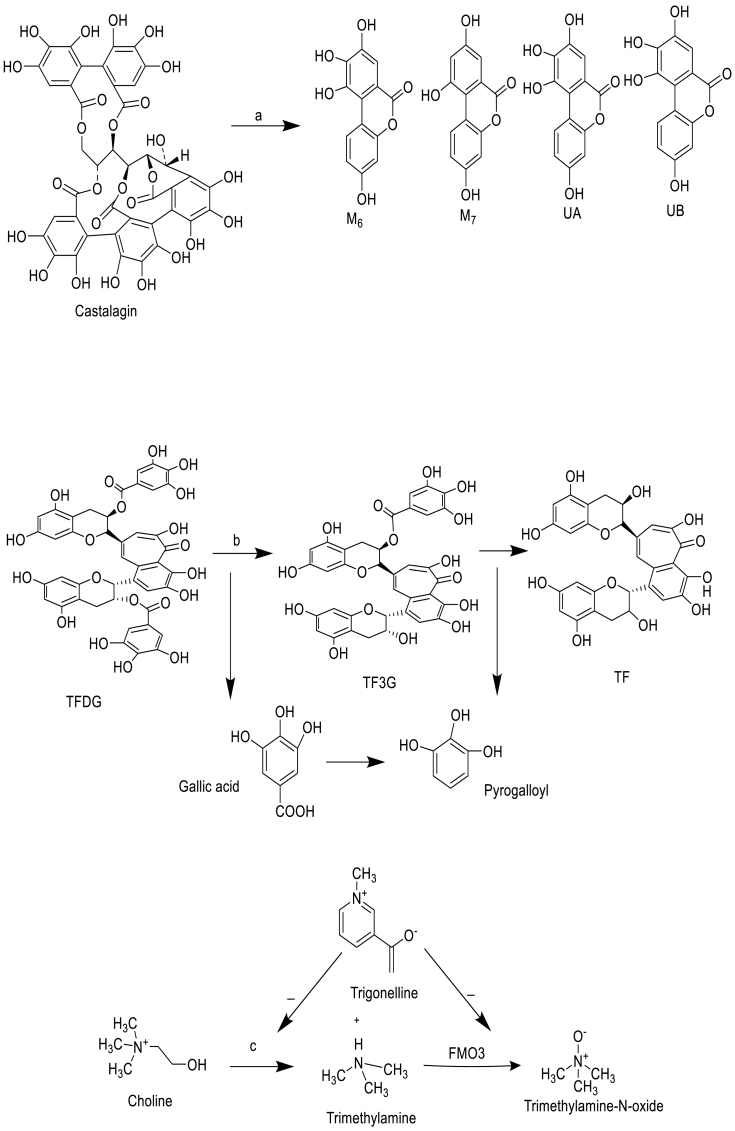

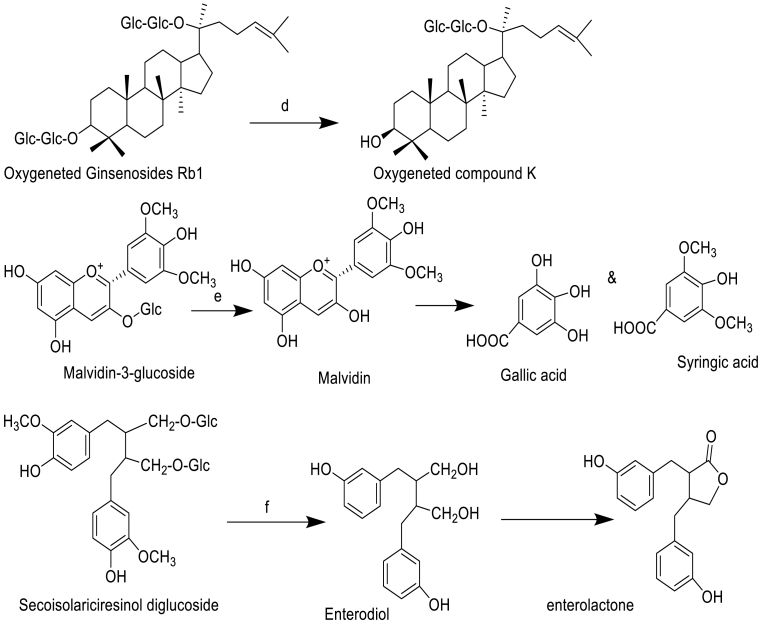
Gut microbiota metabolism of selected NPs with respect to name of microbiota: a, *Gordonibacter urolithinfaciens* and *G. pamelaeae* DSM 19378; b, *Lactobacillus plantarum* 299, *Bacillus subtilis*; c, *Citrobecter freundii and* flavin monooxygenase inhibition; d, *Eubacterium, Clostridiales*; e, *Lactobacillus plantarum*, *Lactobacillus casei*, and *Bifdobacterium lactis*; f, *Peptostreptococcus* and *Eubacterium* species.

The metabolic crosstalk between the host and gut microbiota modulates the pharmacokinetic and pharmacodynamic properties of NPs.^18^ Direct and indirect mechanism of gut microbiota is very much renowned for the promising effect of pharmacokinetic by participating in the direct metabolism of NPs or through its indirect interaction with the host enzymatic system mechanistically. The indirect interaction is facilitated through the production of microbial or mammalian microbial co-metabolites that compete for the metabolism of xenobiotics compounds found in our diet including NPs or act as signaling molecules that influence the host gene expression.^19^ All types of probable metabolic conversions of NPs with specific microbiota are shown in (Fig. 2).

The metabolic fate of ETs molecules with gut microbiota using *in vivo* cultures was nicely reported.^20^ This depends upon a rate-limiting manner with promising yield of microbial metabolites and other derivatives of ellagic acid. Two species of bacteria, *Gordonibacter urolithinfaciens* and *G. pamelaeae* DSM 19378, being able to sequentially metabolize 1 to urolithins M5, M6, and UC (Fig. 2). The species of microbiota metabolizing UA and UB and those responsible for alternative modes of deoxygenation remain unidentified.^20^

Theaflavins (TF), theaflavin-3-gallate (TF3G), theaflavin-3′-gallate (TF3′G), and theaflavin-3, 3′-digallate (TFDG) are the major bioactive polyphenols present in black tea. From fecal microbiota from healthy human volunteers involved in the metabolism of TFDG, and found that TFDG is metabolized to TF, TF3G, TF3′G, gallic acid, and pyrogallol. Moreover, both TF3G and TF3′G are metabolized to TF, gallic acid, and pyrogallol by human microbiota. *Lactobacillus plantarum* 299v and *Bacillus subtilis* (Fig. 2) can metabolize to TFDG.^21^

Trigonelline significantly reduced levels of TMAO and was estimated due to flavin mono-oxygenase (FMO3) enzyme inhibition under *ex vivo* conditions. So, the gut microbiota in choline metabolism and consequently the production of metabolites was causing CVD. The growth of isolated bacteria *C. freundii* strain was checked by Kirby-Bauer disk diffusion susceptibility test in the presence and absence of trigonelline. Further, *in vitro* conversion of trimethylamine (TMA) to trimethylamine oxide (TMAO) through isolated liver FMO3 enzyme was studied and in absence of trigonelline, TMAO (Fig. 2) was formed.^22^

The conversion of ginsenosides (Rb1) in the gastrointestinal tract has been largely studied using *in vitro* and *in vivo* studies. The de-glycosylation reactions occurred by intestinal bacteria *Eubacterium, Clostridiales* via stepwise cleavage of the sugar moieties. The metabolic product of Rb1 i.e. compound K has been reported to have potential antitumor effects and stronger than its parent compound ginsenosides Rb1 and Rd (Fig. 2).

Anthocyanins as anthocyanidin aglycons (cyanidin, pelargonidin, and malvidin) have very low bioavailability so do not appear to undergo extensive metabolism. Large amounts of these compounds enter the colon, where undergo de-glycosylated by gut microbiota. Malvidin-3-glucoside (from grape extracts) with *L. plantarum*, *L. casei* (Fig. 2) under incubation to the formation of syringic, gallic, and *p*-coumaric acids.

Lignans (1,4 diarylbutane structure such as secoisolariciresinol, syringaresinol, and others) metabolism includes glucuronidation and to a lesser degree of sulfation. The biological activity of lignans is related to the activation of these compounds by *Bacteroides and Clostridium* species (in the gut microbiota) to enterolactone and enterodiol (Fig. 2). This transformation of lignans was carried out after de-methylation and de-hydroxylation reactions (carried out by *Peptostreptococcus* and *Eubacterium* species) and probable derivatives reached circulation.^23^

### 2.2 Gut microbiota biotransformation

**Fig. 3:**
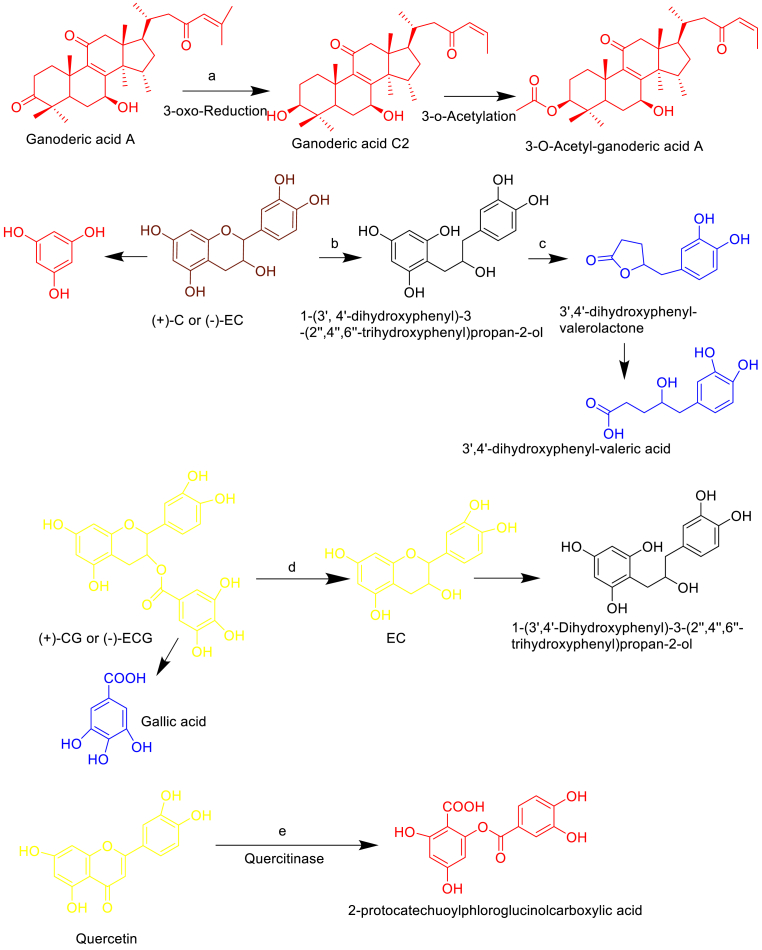

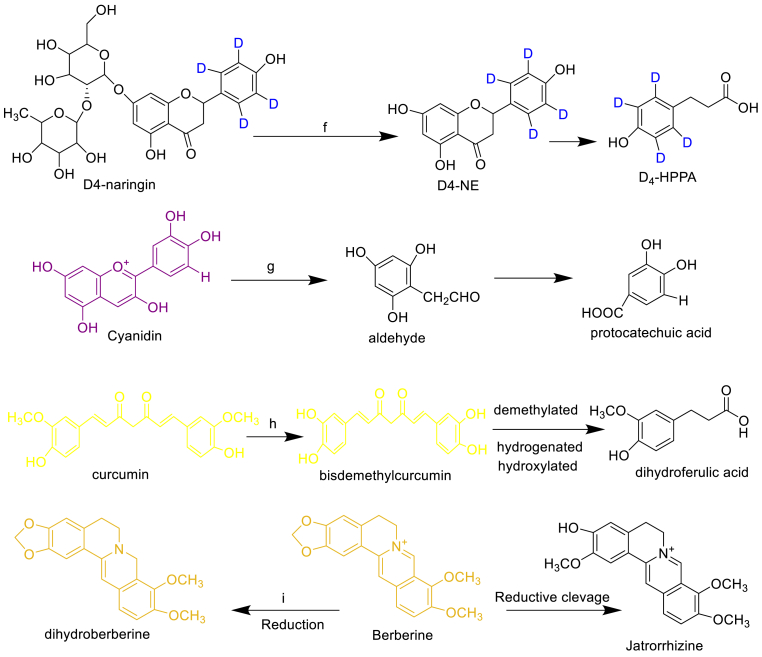
Gut microbiota biotransformation of selected NPs with respect to name of microbiota: a, *Streptomyces* sp. *AI 045*; b, *Eggerthella lenta rK3*; c, *Eggerthella lenta rK3*; d, *Bifidobacterium* and *Clostridium*; e, *Streptomyces urythermus*, *Aspergillus niger*, *A. flavus*, *Penicillium olsonii*, *P. decumbens*; f, *Clostridum* spp., *E. ramulus*; g, *L. plantaurum and L. casei*; h, *Blautia* sp. *Escherichia fergusonii*, *Escherichia coli;* i*, Escherichia coli.*

Some well-known investigations might be substantially inspired by the gut microbiota biotransformation of selected NPs as shown in (Fig. 3).

Although numerous triterpenoids have been identified from the medicinal fungus *G. lucidum*,^24^ few studies have been published on the microbial biotransformation of ganoderma triterpenoids. Gut microbiota biotransformation of ganoderma triterpenoid GA-A and with soil-isolated *Streptomyces* sp. *AI 045*, making it the first case in which a microbe was found to biotransform triterpenoid to its 3-O-acetyl derivative. This is the first time that the acetylation metabolite 3-O-acetyl GA-A has been identified. Although numerous triterpenoids of *G. lucidum* were reported for several biological disorders.^25^

Catechins, revealed that both (+)-C and (–)-EC biotransformed to 1-(3′, 4′-dihydroxyphenyl)-3-(2'',4'',6''-trihydroxyphenyl)propan-2-ol by *Eggerthella lenta rK3*. However, the conversion of (+)- proceeded five times faster than that of (–)-EC. *Flavonifractorplautii* aK2 further converted 1-(3′,4′-dihydroxyphenyl)-3-(2'',4'',6''- trihydroxyphenyl) propan-2-ol to δ-(3′,4′-dihydroxyphenyl)-γ-valerolactone and δ-(3′,4′-dihydroxyphenyl)-γ-valeric acid and other small metabolites. (Fig. 3).^26^

The (+)-catechin gallate (CG) and (–)-epicatechin gallate (ECG), (+)-CG and (–) ECG are the 3-O-gallate products of (+)-C and (–)-EC, respectively. Since (–)-ECG is the major constituent of tea, its biotransformation has extensively been investigated, while the biotransformation of (+)-CG has not been reported. The biotransformation of (–)-ECG is discussed. The first step of microbial metabolism of (–)-ECG is hydrolysis, giving gallic acid and (–)-EC. Gallic acid is metabolized to pyrogallol, and the metabolism of (–)-EC follows that discussed and reported that 13 metabolites were found after incubation of (–)-ECG with human intestinal microbiota (Fig. 3) (*Bifidobacterium strain*)*.* Epicatechin (+)-C, (–)-EC and epigallocathechin gallate (+)-CG and (–) ECG biotransformed to 3-(3-hydroxypjenyl) propionic acid and 5-(3′,4′-dihydroxyphenyl)-γ-valerolactone by *Bifidobacterium* and *Clostridium*.

Flavanones and flavonols in hops are mainly represented by derivatives of naringenin (flavanone) and quercetin (flavonol), and are subjected to microbial transformations in the heterocyclic C ring. Quercetin is converted via oxygenolytic ring-cleavage, catalyzed by flavonol 2, 3- di-oxygenase (*quercetinase*), leading to the formation of 2-protocatechuoylphloroglucinol carboxylic acid (Fig. 3). Quercetinase activity was found in several fungal strains such as *Streptomyces urythermus*, *Aspergillus niger*, *A. flavus*, *Penicillium olsonii* and *P. decumbents*.^27^

In plants, most flavanones are found as glycosides (e.g. D4-naringin) and they are initially de-glycosylated followed by C-ring cleavage with β-glucosidase enzyme responsible microbiota to give D4-NE ([2′, 3′, 5′, 6′-D4] naringenin) and D4-HPPA (3-(4′-hydroxyphenyl)-[2′, 3′, 5′, 6′-D4] propanoic acid). Clostridum strains and *E. ramulus* (Fig. 3) are responsible for this conversion. In the colon, flavone C-multiglucosides undergointestinal absorption without any degradation, translocate to the liver through portal circulation, and then re-circulates intact to the intestine through enterohepatic circulation**.**

Anthocyanins undergo de-glycosylation by bacterial enzymes (e.g. α, l-rhamnosidase and β, d-glucosidase), and then cleavage of C-ring at various sites results in formation of aldehydes (Fig. 3) derived from A-ring and small phenolic acids from B-ring. This report concluded that the first step of the bacterial biotransformation (*L. plantaurum* and *L. casei*) to yield tentative small metabolites. De-carboxylation and de-hydroxylation products of protocatechuic acid, catechol, and phenol, were identified as metabolites.

Tan et al., investigated the biotransformation of three curcuminoids (curcumin, demethoxycurcumin, and bisdemethoxycurcumin) by human fecal microbiota (Fig. 3) using a model *in vitro*. Three main metabolites, including tetrahydrocurcumin, dihydroferulic acid and 1-(4-hydroxy- 3-methoxyphenyl)-2-propanol, were detected through the use of ultrahigh-pressure liquid chromatography (UHPLC), coupled with a linear ion trap mass spectrometer after 24 h of fermentation. It was employed ultra-performance liquid chromatography coupled with quadrupole time of flight mass spectrometry (UPLC-Q-TOF-MS) with automated data analysis software to identify curcumin metabolites produced by human intestinal flora. A total of 23 metabolites were registered and several novel human gut microbiota curcumin metabolic pathways, via demethylation, reduction, hydroxylation, and acetylation, or the combination of these, were revealed. Burapan et al., reported their findings of demethylation as an important metabolism pathway of curcuminoids (a mixture of curcumin (1), demethoxycurcumin (2), and bisdemethoxycurcumin (3), by the human intestinal bacterium *Blautia* sp. MRG-PMF1. It was found that curcumin was converted to two new metabolites, demethylcurcumin and bisdemethylcurcumin through the methyl aryl ether cleavage reaction. Furthermore, Shen et al. studied, the biotransformation of curcuminoids by *Escherichia fergusonii* (ATCC 35469) and two *Escherichia coli* strains (ATCC 8739 and DH10B).^28^

The major problem of berberine with poor oral bioavailability (<5%) and a quiet low concentration in plasma while its metabolites were usually maintained at high plasma concentrations.^29^ Nitroreductases (≥90%, recombinant, expressed in *Escherichia coli*) produced by gut microbiota (Fig. 3) could convert BBR into dihydroberberine (dhBBR), which had an intestinal absorption rate that was five-fold higher than that of BBR. However, an unstable form of dhBBR and would be oxidized to BBR in intestines before the final absorption. This summarizes the role of the gut microbiota biotransformation in regulating the conversion-absorption-reversion process of BBR in the intestine system. In another study, anaerobic cultured gut microbiota of rats could also transform BBR into other metabolites, including berberrubine (BRB), demethyleneberberine (DMB) and jatrorrhizine (JAT). BRB, DMB and JAT possessed higher lipophilicity than BBR, and were of great significance for many therapeutic activities of BBR.^30^^,^^31^

## 3 The experimental perspective of NP metabolites via computational hypothesis approach

**Fig. 4:**
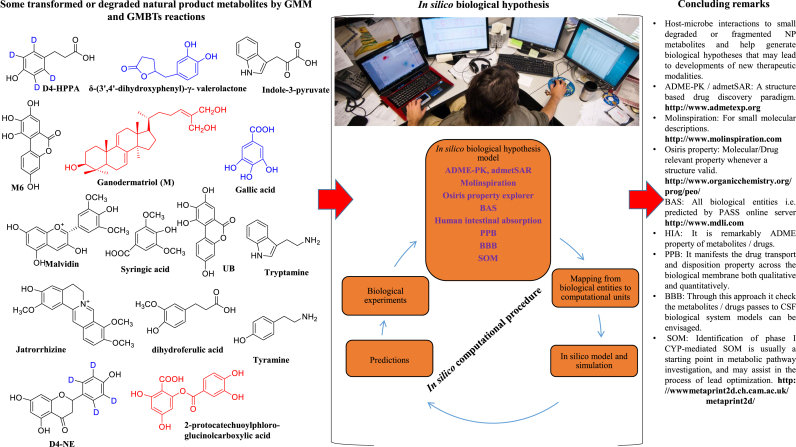
Schematic illustrations of the GMM and GMBTs metabolite undrgoes *in silico* biological hypothesis through computational chemistry to biology models of valid natural product metabolites gives full remarks via biochemoinformatics tools-drug discovery, chemical structure, biological targets, biological activity fingerprint, toxicity profiling, site of metabolism and data visualization to predicts possible chemistry and biology before wet lab experimental procedure.

The discovery of new pharmaceutical drugs is one of the preeminent tasks—scientifically, economically, and socially in biomedical research. Advances in bioinformatics and computational biology have increased productivity at many stages of the drug discovery pipeline. So, several procedures of biochemoinformatics via computational tool or free web server to identify and predicts the significant NP metabolites in terms of the score of specific pharmacological activity by well-known models and databases (Fig. 4). Nowadays these *in silico* computational approaches play a vital role in drug discovery development of natural products via routine experimental analysis in the laboratory and in the future to prevent the exploitation of valuable metabolites and molecules.^32^

### 3.1 In silico biological hypothesis

Such methods have been integrated approach to frequent use in the discovery and optimization of plant, microbe derived molecules and metabolites with affinity to a target, the clarification of absorption, distribution, metabolism, excretion, toxicity properties (ADME-PK models), as well as physicochemical and biological characterization like drug likeliness, biological activity spectrum, ligand-receptors based and metabolic predictions (SOM).^32^ So that utility of drug discovery will become functionalized.

#### 3.1.1 In silico pharmacokinetics

These tools to investigate absorption, distribution, metabolism, excretion, and pharmacokinetics (ADME-PK) properties of new chemical entities (NCEs) i.e. new metabolites are an integral part of the current industrial drug discovery paradigm.^32^

#### 3.1.2 Molinspiration

Molecular descriptors and drug likeliness properties of metabolites and molecules were analyzed by the Molinspiration server (http://www.molinspiration.com), based on Lipinski Rules of five. It states that most “drug like” molecules must have log P ≤ 5, molecular weight ≤500, number of hydrogen bond acceptors ≤10, and number of hydrogen bond donors ≤5. It calculates the molecular properties such as (LogP, number of hydrogen bond donors, acceptors, and polar surface area) with a prediction of bioactivity score with respective drug targets (GPCR ligands), kinase inhibitors, ion channel modulators, enzymes, and nuclear receptors.

#### 3.1.3 Toxicity risk assessment

This assesses the undesirable toxic properties of our metabolites and compounds from Osiris Property Explorer (http://www.organicchemistry.org/prog/peo) was used. It shows in terms of mutagenic, tumorogenic, irritant, reproductive effects, drug-relevant properties [c Log P, Log S (Solubility)], molecular weight, and overall drug-score were calculated with different color codes.

#### 3.1.4 Biological activity spectrum

It describes the intrinsic properties of the compound depends on its structures with high particularities. The set of pharmacological effects, mechanisms of action, and specific toxicities that might be exhibited by a particular compound in its interaction with biological entities are predicted by PASS (http://www.mdli.com). PASS uses Sdffile (.sdf) or MOL file (.mol) formats as an external source of structure and activity data. Their values vary from 0.000 to 1.000. Only those activity types for which Pa > Pi was considered possible.

#### 3.1.5 Human intestinal absorption (HIA)

A large amount of data regarding HIA has been produced rapidly by *in vivo* and *in vitro* experimental assays. Many computational classification and correlation models have been developed to predict the HIA-based data. The most influential substructure patterns and metabolites are recognized by an information gain analysis.

#### 3.1.6 Plasma Protein Binding (PPB)

These models can also be classified as ligand and receptor-based models, as shown in (Fig. 4). The characteristics of small molecules can be directly used for binding sites and affinity prediction, forming some ligand-based HSA binding models.

#### 3.1.7 Blood-brain Barrier (BBB)

Most *in silico* models that are devoted to this aim are based on the assumption that compounds are transported across the BBB by passive diffusion. Lanevskij et al.,^33^ developed a simple QSAR model based on log P, pKa, and fraction unbound on the plasma for log BB prediction, also considering the influence of brain tissue binding by estimating the negative logarithm of the fraction that is unbound in the brain (−log fu, br) with a non-linear ionization-specific model that is based on log P and pKa. As a result, the model demonstrated good predictive power for both internal and external validations.

#### 3.1.8 Metabolism

Currently, metabolism-related prediction models have mainly focused on the following studies: (1) the interaction models of enzymes with xenobiotics/molecules, which were often used to distinguish whether a xenobiotic is a substrate or inhibitor of Cytochrome P450 monooxygenase system, and then to evaluate DDIs; (2) the clearance models of the liver that could quantitatively predict the metabolic stability of xenobiotics.

The interaction of dietary natural compounds (like polyphenols, triterpenoids, polysaccharides and others) and gut microbiota plays a substantial role in human health considerations. To solve this complex category of natural compounds, the genome scale metabolic networks and bioinformatic tool predicts the novel bioactive metabolites through gut microbiota transformations. Recently, AGORA-based REconstruction for Diet Analysis (AGREDA), is thus more agreeable to analyze the role of the human gut microbiota in diet metabolism. This revealed degradation pathways of 209 compounds present in the human diet, mainly phenolic compounds, a family of metabolites highly relevant for human health and nutrition.^34^ At present, very few scientific measures are reported for the qualitative and quantitate analysis of biomarkers in the context of category of natural compounds vs gut microbiota is a key question for balancing in host health.

#### 3.1.9 Metabolite prediction

The purpose of metabolite prediction is to identify the primary metabolites for xenobiotics. This type of study can also provide insight into the mechanisms that are involved in metabolite related toxicity or other pharmacological research. The methods that are available for metabolite prediction can be divided into expert systems and statistical-based methods. Ahlstrom et al.**,**^35^ was used MetaSite, a metabolism site prediction program, to optimize the metabolic stability of celecoxib. Most analogs may retain their inhibitory activities, and their metabolic stabilities toward CYP2C9 were also improved. The consensus score was developed based on five metabolism prediction methods, including MetaPrint2D, SmartCYP, MetaDrug, MetaSite, and SOM.

## 4 Role of gut microbiota vs natural products associated with several diseases

The experimental perspective via wet lab of dietary NPs by gut microbiota modulation nowadays is the novel potential therapeutic target in drug discovery and development approach. These experimental procedures are increasingly applied as a powerful method to identify bioactive compounds from gut microbiota and to identify the microbiota-driven biological mechanism with the rodents-based model.

### 4.1 Metabolic disorders

The gut microbiome actively mediates the pathogenesis in a myriad of metabolic disorders^28^ viz., obesity, CVD, T2DM, and inflammatory bowel diseases. The synergistic effect of isoquercetin and inulin was completed from two metabolically related ways (i) direct prebiotic effect of inulin to improve gut microbiota profiles, and (ii) this improved microbiome with isoquercetin facilitating the rate of absorption and metabolism, thereby mutually enhancing its direct beneficial effects.^36^ The combination diet with mice model also exhibited much abundance of *Faecalibaculum rodentium*, mainly known to produce high quantities of lactic acid as a source of lactate. *F. rodentium* dependent biotransformation reaction to produce small metabolites propionate and butyrate and gut-derived propionate is used in hepatic synthesis of odd-chain fatty acids, which are associated with a reduced risk of T2DM. Supplementation of *G. lucidum* polysaccharide strain S3 (GLPS3) increased the relative abundance of the beneficial bacteria such as lactobacillus, roseburia, and lachnospiraceae. GLPS3 inhibits pancreatitis through microbiota regulation.^37^ Curcumin with antimicrobial activities should be further investigated as novel adjunctive therapies for NAFLD. In addition, NAFLD patients were found with a significant over-representation of Lactobacillus species and some phylum Firmicutes (Lachnospiraceae; genera Dorea, Robinsoniella, and Roseburia), as well as a significant under-representation in phylum Firmicutes (Ruminococcaceae; genus Oscillibacter). Also, a significant elevation of volatile organic compounds (VOC) was observed in their feces.^38^

### 4.2 Gastrointestinal physiology (GIP)

Dietary polyphenols affect the microbiome, resulting in health promotion by the activation of short-chain fatty acids (SCFA) excretion and intestinal immune function.^39^ Curcumin and resveratrol also decrease the *Firmicutes:Bacteroidetes*^40^ ratio and have anti-inflammation and anti-carcinogenesis effects by modifying colonic microbial ecology in animal experiments. Procyanidins (flavan-3-ol moieties) and are subject to metabolization by gut microbiota in experimental animals disease models like cancer, heart disease, and diabetes. The metabolic products of procyanidins are majorly absorbed in the system and have shown a variety of pharmacological activities *in vitro* and experimental animals.^41^ In addition to these prebiotic-like effects, mainly preclinical studies indicate that these polyphenols can shape gut microbiota by increasing the *Firmicutes:Bacterioides* ratio and by positively influencing the abundance of certain microbial species that may confer health beneficial effects to the human host, such as *Akkermansia muciniphila*, *Faecalibacterium prausnitzii,* and *Roseburia* sp.^42^

### 4.3 Cancer

Modulation of gut microbiota by glycyrrhizinic acid (GA) suppresses HFD-enhanced pre-metastatic niche formation, recolonizes the *Desulfovibrio vulgaris* and *Clostridium sordellii*, and prevents the metastasis of 4T1 breast cancer and B16F10 melanoma.^43^ Colonization with lignin-metabolizing microbial community protected germ-free rats from 7, 12-dimethylbenz(a)anthracene-induced cancer and tumor. Both urolithins A and B, the most representative microbial metabolites of dietary ETs by *Gordonibacter urolithinfaciens* and *G. pamelaeae* DSM 19378, have shown oestrogenic activity in a dose-dependent manner, without antiproliferative or toxic effects towards MCF-7 breast cancer cells.

### 4.4 Gut-brain disorders

Curcumin, a nontoxic, naturally occurring polyphenol, has been recently proposed for the management of neurological and neurodegenerative diseases. The experimental study indicated that curcumin favors brain health by modulating specific pathways such as the PI3K/Akt (Phosphatidylinositol 3-kinase/Protein Kinase B) pathway, MAPK (Mitogen-Activated Protein Kinase)/Akt pathways, Akt/Nrf2 (Nuclear factor-E2-related factor 2), and AMP (AMP-activated protein) kinase pathway.^44^ The blackberry anthocyanins can prevent some of the features of HFD-induced dysbiosis. In addition, anthocyanin-induced changes in the gut microbiota composition are related to their anti-neuroinflammatory properties. LW-AFC—*Liuwei Dihuang* a saccharide-enriched fraction, some small molecules such as rhein (the main rhubarb ingredient) was also demonstrated to improve the recognition memory capability (Alzheimer's disease) in HFD-fed mice through increasing the abundance of Lactobacillus and Bifidobacterium.^45^

## 5 Conclusion and future directions

Ethnopharmacological relevance's point of view, personalized dietary NPs have been fewer documented and used for several medical disorders like asthma, immunomodulatory, arthritis, cough, malaria, COPD, CVD, diabetes, and cancer. Regulation and restoring the aesthetic value of NPs concerns nowadays look interesting with special remarks on the diversity of human gut microbiota populations. Because a specific population of gut microbiota and associated reactions have the caliber to transform ingested NPs to most bioactive metabolites i.e. prone to biology for specific ailments, which regulates the dysbiosis to eubiosis status of gut microbiota niche. These metabolites are nowadays the most promising for phytopharmacology considerations with specified animal models through gut microbiota metabolism i.e. phase I and II reactions. Health benefiting bacteria *F/B*, Akkermansia, Bifidobacterium, Lactobacillus, and Faecalibacterium could be the most important targets for the treatment in case of illness associated with dysbiosis. This approach channelizes host physiology with ingested bioactive and efficient NPs via regulating gut microbiota composition and plays a vital role in therapeutic significance in the upcoming era.

So, over here one of the most promising tools i.e. Bio-Chemoinformatics applied for degraded molecules that procured from gut microbiota modulation with several biochemical reactions. This tool gives the prediction of the biological activity of degraded molecules and plays a vital role in the preliminary biological hypothesis before the rodents-based experimental model. This gut microbiota produced NP metabolites whose biological significances predicts by *in silico* Bio-Chemoinformatics based models. This report gives keen information to harnessing the dietary natural product-metabolites versus host-microbiota interplay disorders with *in silico* bioactivity consideration. Which leads to a better understanding the experimental and computational pharmacology. Hopefully, this combined approach surely gives positive sound for the improvement of host health and prevention or mitigation of several kinds of ailments.

## Novelty

- Gut microbiota might be a potential new territory for drug targeting, especially for dietary natural products
- Gut microbiota vs dietary natural product metabolism and biotransformation to potential metabolite implications to several type of ailments
- The experimental perspective of NP metabolites via computational hypothesis approach
- Role of gut microbiota vs dietary natural products associated in several diseases

## Declaration of competing interest

The authors have no conflict of interest.

## List of abbreviations

ADME-PK: Absorption Distribution Metabolism Excretion and Pharmacokinetic
Akt/Nrf2: Protein kinase B/Nuclear factor-E2-related factor 2
AMP: AMP-activated protein pathway
BBB: Blood Brain Barrier
BBR: Berberine
BRB: Berberubine
dhBBR: Dihydroberberine
CVD: Cardiovascular disorders
CYP450s: Cytochrome P450 monooxygenase system
DMB: Demethyleneberberine
ETs: Ellagitannins
GA: Glycyrrhizinic acid
GIP: Gastrointestinal physiology
GLPS3: *G. lucidum* polysaccharide strain S3
GMBTs: Gut microbiota biotransformation
GMM: Gut microbiota metabolism
GPCR: G-protein coupled receptor
HIA: Human Intestinal absorption
HPLC: High Performance Liquid Chromatography
HAS: Human serum albumin
JAT: Jatrorrhizine
log BB: log blood brain
MAPK: Mitogen-Activated Protein Kinase)
NAFLD: Nonalcoholic fatty liver disease
NCEs: New chemical entities
NPs: Natural Products
PDGFRβ: Platelet-derived growth factor receptor-beta
PI3K/Akt: Phosphatidylinositol 3-kinase/Protein Kinase B
PPB: Plasma Protein Binding
SARS-CoV-2: Severe Acute Respiratory Syndrome Coronavirus 2
SOM: Site of metabolism
T2DM: Type-2 diabetes mellitus
VEGFR2: Vascular endothelial growth factor receptor-2
