# Supplementary material for: Role of gut microbiota metabolism and biotransformation on dietary natural products to human health implications with special reference to biochemoinformatics approach
Source: J Tradit Complement Med. 2022 Apr 14;13(2):150–60. doi: 10.1016/j.jtcme.2022.03.005 (PMC10037058; doi:10.1016/j.jtcme.2022.03.005)
Supplement: Supplementary materials Table 1 and Table 2 [file mmc1.docx]

**Supplementary Table 1.**

Human microbiota composition of the gut extensively studied body sites. Interestingly, the gut microbiota has the highest microbial diversity.

| **Human microbial habitat** | **Human microbiota (10 times more microbial than human cells: 10^14^ vs 10^13^)**  **Phyla and their relative abundance (%) with well-renowned genera** | | **Number of species** | **Reference** |
| --- | --- | --- | --- | --- |
| Gut | **More abundance**  *Firmicutes* (64)  *Bacteriodetes* (27.8)  *Proteobacteria,*(2.1)  *Verrumicrobia* (<1)  *Actinobacteria* (8.2)  *Archaea* (<1)  *Euryarchaeota* | *Ruminococcus, Clostridium*, *Lactobacillus* (other strains); butyrate producers: *Eubacterium*, *Faecalibacterium,* and *Roseburia*;  *Bacteroides*, *Prevotella,* and *Xylanibacter*  *Escherichia, Desulfovibrio*  *Akkermansia*  *Collinsella, Bifidobacterium*  *Methanobrevibacter* | >1000 | ^1,2^ |
|  | **Less abundance**  Cyanobacteria (0.2) Fusobacteria (0.2) Lentisphaerae (0.2)  Spirochaetes (0.2)  Saccharibacteria (2) |  | <100 (few species) | ^3–5^ |

**Supplementary Table 2.**

Section of eubiosis/dysbiosis imbalance associated disease with the variability of gut microbiota.

| **Disease** | **Bacterial changes**  **Decrease Increase** | | **Reference** |
| --- | --- | --- | --- |
| Colorectal cancer | *Fusobacterium*  *Ruminococcus* | *E. coli*  *E. coli*  *Sphingomonas* | ^6^  ^7^ |
| Breast cancer | *S. yanoikuyae*  *Laptococcus*  *Streptococcus* | *M. radiotolerans*  *E. coli*  *S. epidermidis* | ^8^  ^9^ |
| T2D | *Eubacterium*  *Firmicutes* | *Lactobacillus species*  *Bacteriodetes*  *A. muciniphila*  *Desulphovibrio sp.*  *Betaproteobacteria* | ^10^ |
| Obesity | *Bifidobacteria*  *C. perfringens*  *Rominococcus*  *Allobaculum* | *Halomonas*  *Sphingomonas*  *L. reuteri*  *L. gasseri* | ^11^  ^12^ |
| Allergies / Asthma | Roseburia  Faecalibacterium  Firmicutes/SMB53  Ruminococcus  *F. prausnitzii* | *E. coli*  *Clostridium spp.*  *Citrobacter*  *Proteus*  *Klebsiella*  *Escherichia coli* | ^13^  ^14^ |
| CVD / Atherosclerosis  Heart failure | *Bacteroides*  *Bacteroides*  *E. ventriosum* | *Prevotella*  *Streptococcus*  *Escherichia coli*  *Escherichia coli*  *K. penumoniae*  *S. viridans*  *L. rhamnosus GR-1*  *S. boulardii* | ^15^  ^16^  ^17^  ^18^  ^19^ |
| GIP / IBD  Crohn’s disease | *C. leptum*  *Erysipelotrichales Bacteroidales*  *Clostridiales*  *Clostridia*  *Erysipelotrichia*  *Ruminococcaceae Christensenellaceae Erysipelotrichaceae* | *Enterobacteriaceae*  *Pasteurellacaea*  *Veillonellaceae*  *Fusobacteriaceae*  *Negativicutes*  *Bacilli (obligately aerobic) Veillonellaceae, Lactobacillaceae, Acidaminococcaceae*  *Streptococcaceae Peptostreptococcaceae* | ^20^  ^21^ |
| Autism  Depression  PD  AD | *Bacteriodetes*  *Lactobacillus rhamnosus*  *L. helveticus*  *Bifidobacterium longum*  *B. breve*  *B. infantis*  *Coprococcus*  *Faecelibacterium spp.*  *Firmicutes* | *Lactobacillus spp.*  *Bacteriodetes* | ^22^  ^23^  ^24^  ^25^ |

References.

1. Tremaroli V, Bäckhed F. Functional interactions between the gut microbiota and host metabolism. *Nature*. 2012;489(7415):242-249. doi:10.1038/nature11552

2. Zoetendal EG, Rajilic-Stojanovic M, de Vos WM. High-throughput diversity and functionality analysis of the gastrointestinal tract microbiota. *Gut*. 2008;57(11):1605-1615. doi:10.1136/gut.2007.133603

3. Zhou Z, Chen J, Yao H, Hu H. Fusobacterium and Colorectal Cancer. *Front Oncol*. 2018;8. doi:10.3389/fonc.2018.00371

4. Fujio-Vejar S, Vasquez Y, Morales P, et al. The gut microbiota of healthy chilean subjects reveals a high abundance of the phylum Verrucomicrobia. *Front Microbiol*. 2017;8. doi:10.3389/fmicb.2017.01221

5. Schmiedel D, Epple H-J, Loddenkemper C, et al. Rapid and accurate diagnosis of human intestinal spirochetosis by fluorescence in situ hybridization. *J Clin Microbiol*. 2009;47(5):1393-1401. doi:10.1128/JCM.02469-08

6. Louis P, Hold GL, Flint HJ. The gut microbiota, bacterial metabolites and colorectal cancer. *Nat Rev Microbiol*. 2014;12(10):661-672. doi:10.1038/nrmicro3344

7. Richard ML, Liguori G, Lamas B, et al. Mucosa-associated microbiota dysbiosis in colitis associated cancer. *Gut Microbes*. 2018;9(2):131-142. doi:10.1080/19490976.2017.1379637

8. Xuan C, Shamonki JM, Chung A, et al. Microbial dysbiosis is associated with human breast cancer. Takabe K, ed. *PLoS One*. 2014;9(1):e83744. doi:10.1371/journal.pone.0083744

9. Urbaniak C, Gloor GB, Brackstone M, Scott L, Tangney M, Reid G. The microbiota of breast tissue and its association with breast cancer. Goodrich-Blair H, ed. *Appl Environ Microbiol*. 2016;82(16):5039-5048. doi:10.1128/AEM.01235-16

10. Aw W, Fukuda S. Understanding the role of the gut ecosystem in diabetes mellitus. *J Diabetes Investig*. 2018;9(1):5-12. doi:10.1111/jdi.12673

11. Abenavoli L, Scarpellini E, Colica C, et al. Gut Microbiota and Obesity: A Role for Probiotics. *Nutrients*. 2019;11(11):2690. doi:10.3390/nu11112690

12. Boudry G, Hamilton MK, Chichlowski M, et al. Bovine milk oligosaccharides decrease gut permeability and improve inflammation and microbial dysbiosis in diet-induced obese mice. *J Dairy Sci*. 2017;100(4):2471-2481. doi:10.3168/jds.2016-11890

13. Chiu C-Y, Chan Y-L, Tsai M-H, Wang C-J, Chiang M-H, Chiu C-C. Gut microbial dysbiosis is associated with allergen-specific IgE responses in young children with airway allergies. *World Allergy Organ J*. 2019;12(3):100021. doi:10.1016/j.waojou.2019.100021

14. Abdurasulova IN, Tarasova EA, Matsulevich A V, et al. [Changes in the qualitative and quantitative composition of gut microbiota in rats during experimental allergic encephalomyelitis]. *Ross Fiziol zhurnal Im IM Sechenova*. 2015;101(11):1235-1249.

15. Koeth RA, Wang Z, Levison BS, et al. Intestinal microbiota metabolism of l-carnitine, a nutrient in red meat, promotes atherosclerosis. *Nat Med*. 2013;19(5):576-585. doi:10.1038/nm.3145

16. Jie Z, Xia H, Zhong S-L, et al. The gut microbiome in atherosclerotic cardiovascular disease. *Nat Commun*. 2017;8(1):845. doi:10.1038/s41467-017-00900-1

17. Kazemian N, Mahmoudi M, Halperin F, Wu JC, Pakpour S. Gut microbiota and cardiovascular disease: opportunities and challenges. *Microbiome*. 2020;8(1):36. doi:10.1186/s40168-020-00821-0

18. Tun HM, Leung FC, Cheng KM. Role of gut microbiota in cardiovascular disease that links to host genotype and diet. in: *The gut microbiome - implications for human disease*. InTech; 2016. doi:10.5772/64636

19. de Moraes ACF, Fernandes GR, da Silva IT, et al. Enterotype may drive the dietary-associated cardiometabolic risk factors. *Front Cell Infect Microbiol*. 2017;7. doi:10.3389/fcimb.2017.00047

20. Stange EF, Schroeder BO. Microbiota and mucosal defense in IBD: an update. *Expert Rev Gastroenterol Hepatol*. 2019;13(10):963-976. doi:10.1080/17474124.2019.1671822

21. Alam MT, Amos GCA, Murphy ARJ, Murch S, Wellington EMH, Arasaradnam RP. Microbial imbalance in inflammatory bowel disease patients at different taxonomic levels. *Gut Pathog*. 2020;12(1):1. doi:10.1186/s13099-019-0341-6

22. Tomova A, Husarova V, Lakatosova S, et al. Gastrointestinal microbiota in children with autism in Slovakia. *Physiol Behav*. 2015;138:179-187. doi:10.1016/j.physbeh.2014.10.033

23. Messaoudi M, Violle N, Bisson J-F, Desor D, Javelot H, Rougeot C. Beneficial psychological effects of a probiotic formulation ( Lactobacillus helveticus R0052 and Bifidobacterium longum R0175) in healthy human volunteers. *Gut Microbes*. 2011;2(4):256-261. doi:10.4161/gmic.2.4.16108

24. Unger MM, Spiegel J, Dillmann K-U, et al. Short chain fatty acids and gut microbiota differ between patients with Parkinson’s disease and age-matched controls. *Parkinsonism Relat Disord*. 2016;32:66-72. doi:10.1016/j.parkreldis.2016.08.019

25. Vogt NM, Kerby RL, Dill-McFarland KA, et al. Gut microbiome alterations in Alzheimer’s disease. *Sci Rep*. 2017;7(1):13537. doi:10.1038/s41598-017-13601-y
